# Modulation of the Intestinal Microbiota Alters Colitis-Associated Colorectal Cancer Susceptibility

**DOI:** 10.1371/journal.pone.0006026

**Published:** 2009-06-24

**Authors:** Joshua M. Uronis, Marcus Mühlbauer, Hans H. Herfarth, Tara C. Rubinas, Gieira S. Jones, Christian Jobin

**Affiliations:** 1 Department of Medicine and Center for Gastrointestinal Biology and Disease, University of North Carolina at Chapel Hill, Chapel Hill, North Carolina, United States of America; 2 Department of Pathology and Laboratory Medicine, University of North Carolina at Chapel Hill, Chapel Hill, North Carolina, United States of America; 3 Department of Pharmacology, University of North Carolina at Chapel Hill, Chapel Hill, North Carolina, United States of America; 4 Biological Biomedical Sciences Program, University of North Carolina at Chapel Hill, Chapel Hill, North Carolina, United States of America; Charité-Universitätsmedizin Berlin, Germany

## Abstract

It is well established that the intestinal microbiota plays a key role in the pathogenesis of Crohn's disease (CD) and ulcerative colitis (UC) collectively referred to as inflammatory bowel disease (IBD). Epidemiological studies have provided strong evidence that IBD patients bear increased risk for the development of colorectal cancer (CRC). However, the impact of the microbiota on the development of colitis-associated cancer (CAC) remains largely unknown. In this study, we established a new model of CAC using azoxymethane (AOM)-exposed, conventionalized-*Il10^−/−^* mice and have explored the contribution of the host intestinal microbiota and MyD88 signaling to the development of CAC. We show that 8/13 (62%) of AOM-*Il10^−/−^* mice developed colon tumors compared to only 3/15 (20%) of AOM- wild-type (WT) mice. Conventionalized AOM-*Il10^−/−^* mice developed spontaneous colitis and colorectal carcinomas while AOM-WT mice were colitis-free and developed only rare adenomas. Importantly, tumor multiplicity directly correlated with the presence of colitis. *Il10^−/−^* mice mono-associated with the mildly colitogenic bacterium *Bacteroides vulgatus* displayed significantly reduced colitis and colorectal tumor multiplicity compared to *Il10^−/−^* mice. Germ-free AOM-treated *Il10^−/−^* mice showed normal colon histology and were devoid of tumors. *Il10^−/−^*; *Myd88^−/−^* mice treated with AOM displayed reduced expression of *Il12p40* and *Tnfα* mRNA and showed no signs of tumor development. We present the first direct demonstration that manipulation of the intestinal microbiota alters the development of CAC. The TLR/MyD88 pathway is essential for microbiota-induced development of CAC. Unlike findings obtained using the AOM/DSS model, we demonstrate that the severity of chronic colitis directly correlates to colorectal tumor development and that bacterial-induced inflammation drives progression from adenoma to invasive carcinoma.

## Introduction

The ability to mount an inflammatory response following injury or exposure to foreign organisms is vital for host homeostasis and survival. However, a persistently heightened immune response such as that observed in chronic inflammatory disorders severely impairs host organ function, ultimately resulting in disease [1]. A major risk associated with chronic inflammation is increased likelihood of cancer development [2]–[4]. Crohn's disease (CD) and ulcerative colitis (UC) collectively termed inflammatory bowel disease (IBD) is the quintessential example in which chronic inflammation translates to increased cancer risk [2]. The cumulative incidence of colorectal cancer in IBD patients ranges from 7.6% to 18.4%, 30 years post-diagnosis [5]–[8]. Furthermore, epidemiological data suggest that the duration and severity of chronic colitis represent significant risk factors for colitis-associated colon cancer (CAC) [9]–[11]. Although the etiology of IBD remains to be elucidated, animal model-based studies indicate that the host intestinal microbiota triggers an immune response that is requisite for the onset of disease [12]–[15]. This uncontrolled immune response likely represents a defect in one or more immunosuppressive mechanisms intended to provide tolerance to the host intestinal microbiota, resulting in the over-production of pro-inflammatory mediators [12], [16]

The concept of a balanced immunosuppressive response aimed at regulating the microbiota may be best illustrated in interleukin-10 knock-out (*Il10^−/−^*) mice. These mice, which exhibit intolerance to their intestinal microbiota develop spontaneous colitis as the result of microbial-induced activation of effector T cells [17]–[19].

The human colon harbor's as many as 36,000 bacterial species amounting to over 100 trillion aerobic and anaerobic bacteria [20], [21]. As a product of our co-evolution with bacteria, a communication system has developed that allow us to regulate one another, thereby maintaining intestinal homeostasis [21]. An integral role in the cross-talk that occurs between the human intestine and its resident microbiota is performed by innate bacterial sensors known as pattern recognition receptors (PRRs) [21]. Two main classes of PRRs have been shown to regulate communication between the intestinal epithelium and the microbiota. Toll-like receptors (TLRs) and Nod-like receptors (NLRs) serve to alert the host to the presence of bacteria in the extracellular and intracellular spaces respectively [22], [23]. Using the azoxymethane (AOM)/DSS model of CAC, Fukata and co-workers showed that TLR4 participates in the development of colorectal cancer [24]. Although interesting, this study has not directly addressed the impact of the microbiota in CAC development. Additionally, the AOM/DSS model of CAC appears to show a dissociation between the severity of intestinal inflammation and cancer development. Clearly, additional investigation of the impact of bacteria on development of CAC is needed.

In this study, we utilized germ-free and gnotobiotic technology to modulate the content of the intestinal microbiota to determine the direct impact of bacteria on the development of CAC. We demonstrate that AOM-treated *Il10^−/−^* mice develop CAC in the presence of colitogenic bacteria whereas germ-free mice remain disease-free. The presence of colitis directly correlates with tumor multiplicity and acts as a promoter of colorectal cancer. Additionally, AOM-treated *Il10^−/−^*; *Myd88^−/−^* mice failed to develop colorectal tumors, indicating that bacterial signaling through the TLR/MyD88 system is required for development of CAC.

## Results

### Bacterial-mediated colitis enhances colorectal tumorigenesis

**Figure 1:**
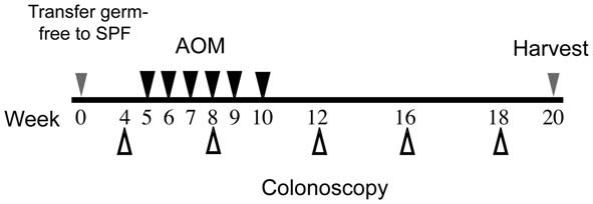
Experimental timeline for AOM-induced colitis-associated colon tumorigenesis and analysis. Germ free mice were transferred to SPF conditions and allowed to acclimate for 5 weeks. Mice were given AOM injections once a week for 6 weeks (black arrowheads). Development of colitis and tumor formation was monitored by colonoscopy from weeks 4 to 18 after transfer from germ free to SPF conditions (white arrowheads). Mice were sacrificed between 18 and 20 weeks and tissues processed for histological and mRNA expression analysis.

**Figure 2:**
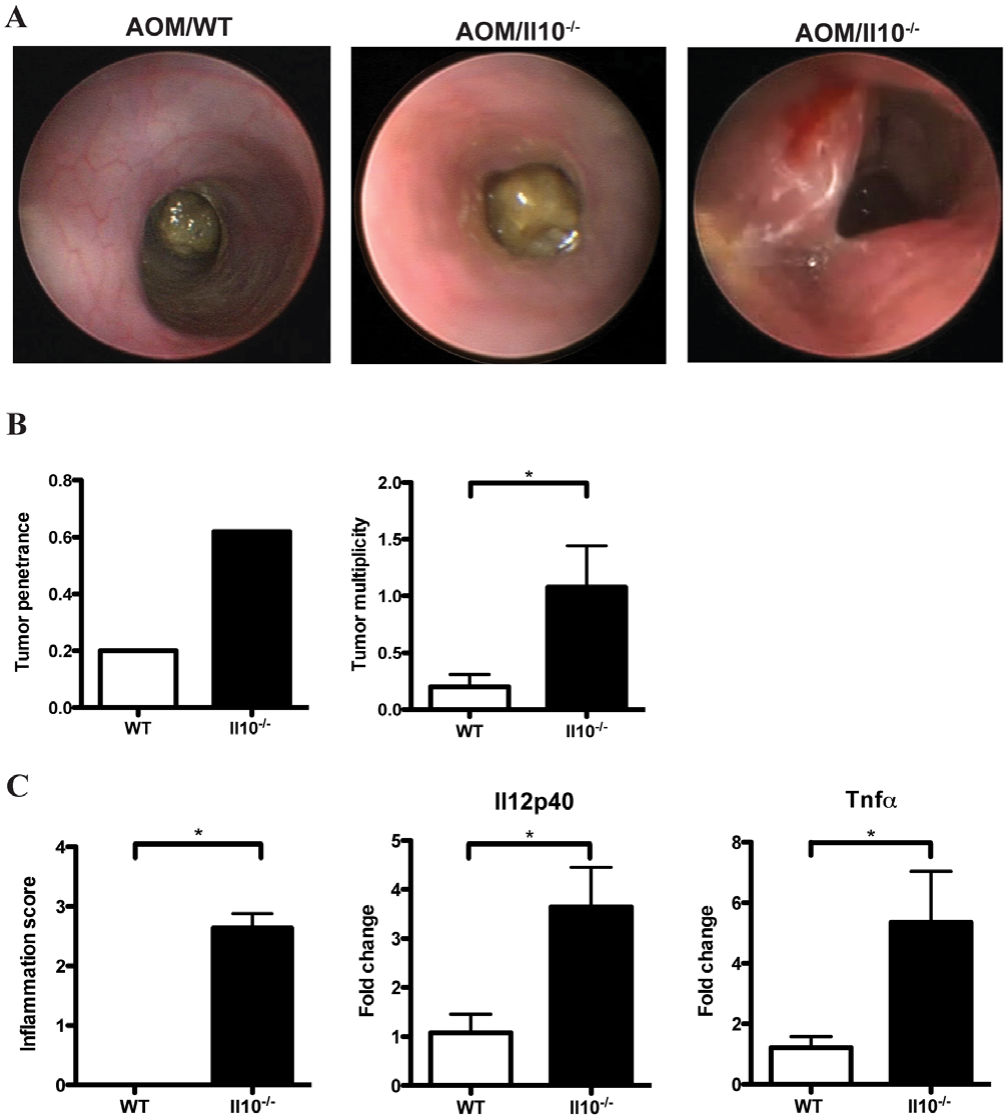
Analysis of tumor induction and inflammation. A. Representative examples of WT (left panel) and *Il10^−/−^* colons (middle and right panels) from AOM-treated mice 16 weeks after transfer to SPF conditions. B. Tumor penetrance and multiplicity in WT and *Il10^−/−^* mice (left and right panels) (p = 0.034). C. WT and *Il10^−/−^* colon inflammation scores, distal colon *Il12p40* and *Tnfα* mRNA levels after 18–20 weeks under SPF conditions (p = 0.026).

To investigate the role of bacterial-mediated colitis in the onset of colorectal carcinogenesis, conventionalized WT and *Il10^−/−^* mice were administered a regimen of AOM and monitored *in vivo* for signs of colitis and tumor development by colonoscopy at defined intervals (Fig. 1). After 16 weeks, WT mice showed no evidence of macroscopic inflammation and displayed a semi-translucent mucosa with well-defined vascularization associated with a healthy colon (Fig. 2A, left panel). In contrast, *Il10^−/−^* mice exhibited mucosal thickening and loss of apparent vasculature associated with macroscopic intestinal inflammation (Fig. 2A, middle panel). Macroscopic lesions compatible with tumors were observed in conventionalized *Il10^−/−^* mice at week 16 (Fig. 2A, right panel). To further study these lesions, histological analysis of colonic tissues was performed on WT and *Il10^−/−^* mice. We confirmed that the macroscopic lesions observed in *Il10^−/−^* mice were tumors that developed in the inflamed colon. As shown in Fig. 2B, AOM-treated *Il10^−/−^* mice showed a dramatic increase in tumor penetrance and multiplicity compared to WT mice. In our study, 8/13 (62%) of *Il10^−/−^* mice developed colon tumors compared to only 3/15 (20%) of WT mice. Furthermore, tumor multiplicity was 5-fold higher in *Il10^−/−^* mice with an average of 1- compared to only 0.2 tumors in WT mice (Fig. 2B) (p = 0.034). Of note, tumor development in *Il10^−/−^* mice was associated with enhanced intestinal inflammation as determined by histological analysis (Fig. 2C) (p<0.0001). Colonic expression of *IL12p40* and *TNFα* mRNA were significantly elevated in *Il10^−/−^*- compared to WT mice (Fig. 2C).

To further investigate the link between bacterial-induced colitis and development of tumors, we performed linear regression analysis, comparing histological inflammation score against tumor number. Pearson correlation analysis revealed a strong positive correlation between inflammation and colon tumor development (p = 0.0028). These data suggest that chronic colitis enhances the progression of early transformation events initiated by AOM, perhaps through establishing a microenvironment more supportive of cellular proliferation.

### Chronic colitis enhances AOM-induced colorectal tumor progression

**Figure 3:**
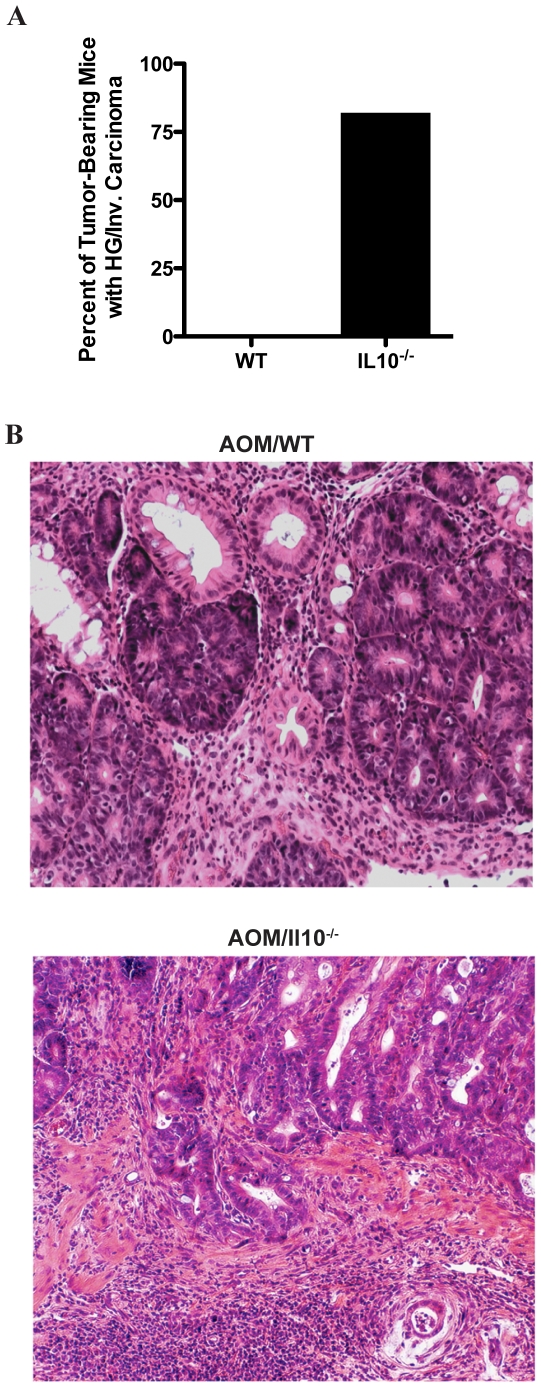
*Il10^−/−^* mice exhibit accelerated tumor progression. A. Percent of tumor-bearing WT and *Il10^−/−^* mice with high-grade or invasive carcinoma (upper panel). Representative low-grade adenoma observed in WT mice (middle panel). Representative invasive carcinoma observed in *Il10^−/−^* mice (lower panel).

Considering the dramatic increase in susceptibility to colon tumor development, we hypothesized that mice with chronic colitis may also demonstrate heightened susceptibility to advanced tumor progression. Histological evaluation was performed and tumors were classified as either as low- or high-grade dysplasia or as invasive carcinoma. Seven of eight (88%) of tumor bearing AOM/*Il10^−/−^* mice developed high-grade or invasive carcinoma while none of the 3 tumors observed in WT mice showed evidence of advanced histological stage (Fig. 3A). Representative examples of colon tumor histology observed in AOM-treated WT and *Il10^−/−^* mice are shown in figure 3B. These data suggest that chronic colitis not only predisposes to increased susceptibility to AOM-induced colorectal tumor development but also enhances the tumors oncogenic potential, thereby promoting progression to more advanced stages.

### NFκB activity and cellular proliferation in tumors from *Il10^−/−^* mice

**Figure 4:**
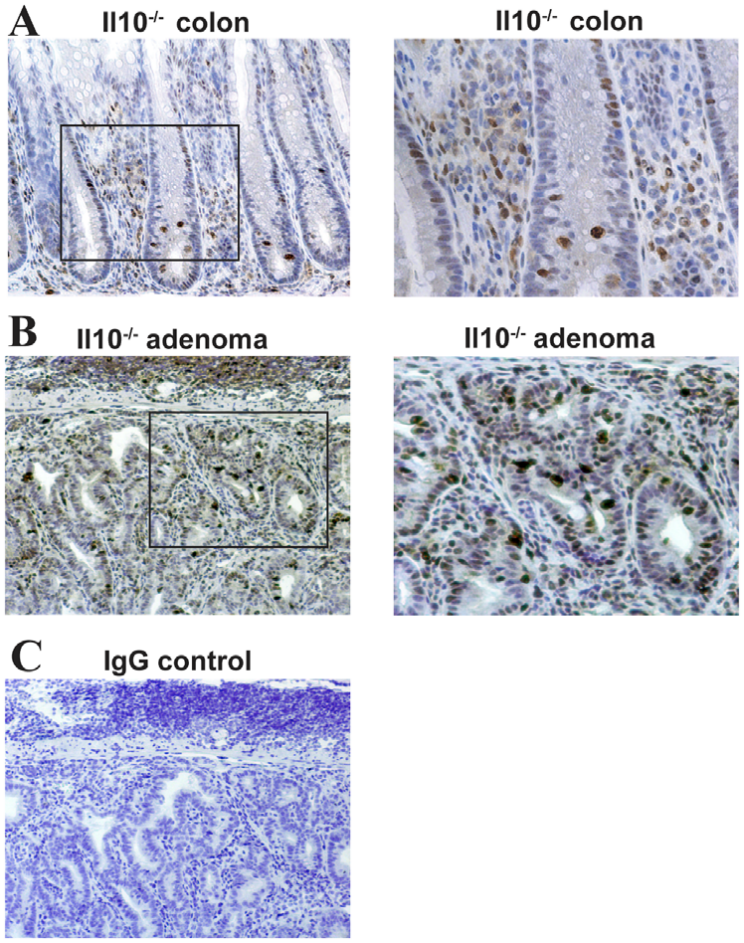
*Il10^−/−^* mice develop inflammation displaying active Rel A (NFkB) signaling. A. Phosphorylated-Rel A immunostaining of normal *Il10^−/−^* colon tissue. B. Enlargement of Rel A positive immune infiltrate in A. C. Phosphorylated Rel A immunostaining in *Il10^−/−^* adenoma. D. Enlargement of adenoma in C. E. Rabbit IgG negative control.

NFκB signaling plays a critical role in promoting bacterial-induced colitis in *Il10^−/−^* mice [25], [26]. To verify the presence of activated Rel A (NFκB)-signaling in the AOM/*Il10^−/−^* model of CAC, we assessed the expression/localization of phosphorylated RelA (S276) in *Il10^−/−^* colon tissues by immunohistochemistry (IHC). Colons from *Il10^−/−^* mice showed abundant phosphorylated RelA positive infiltrating immune cells (Fig. 4A). Furthermore, the presence of numerous phosphorylated RelA positive immune- and intestinal epithelial cells were observed in adenomas from *Il10^−/−^* mice (Fig. 4B).

**Figure 5:**
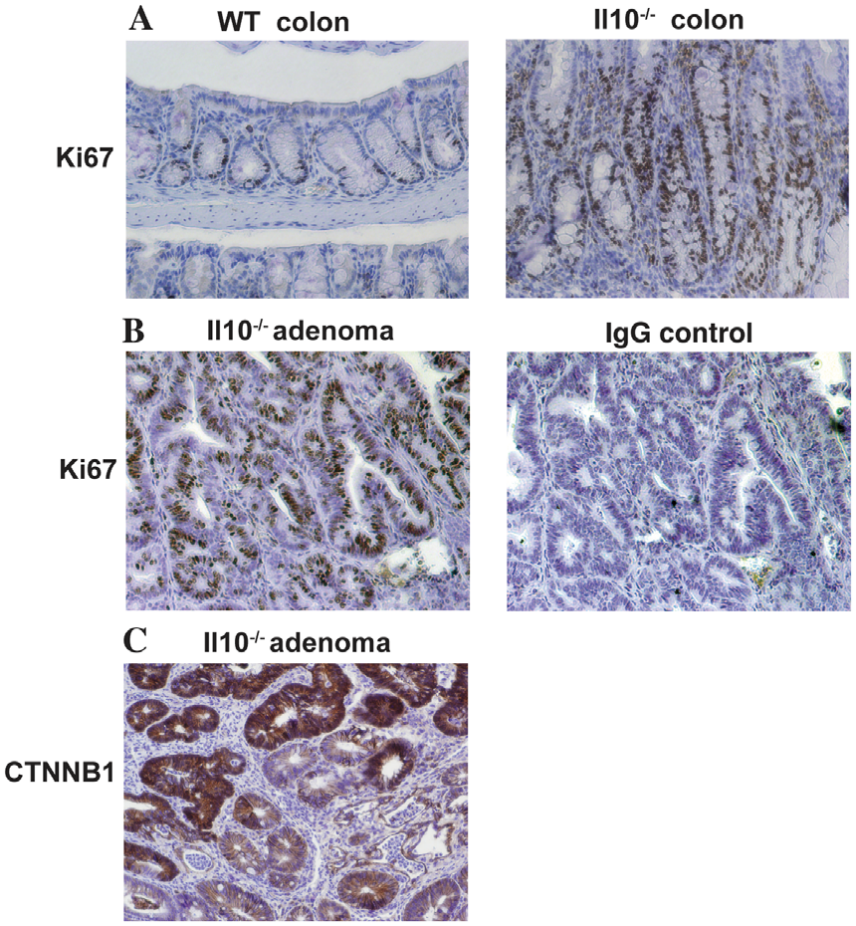
*Il10^−/−^* normal colon and adenomas exhibit increased cell proliferation. A. Ki67 positive cells are restricted to crypt bases in normal WT colon. Normal colons from *Il10^−/−^* mice exhibit elongated crypts with expanded Ki67 positive staining. B. Representative adenoma from *Il10^−/−^* mouse shows increased Ki67 positive staining (left panel). Rabbit IgG negative control (right panel). C. *Il10^−/−^* adenoma shows positive CTNNB1 staining.

Due to its role in promoting cellular proliferation and survival, we evaluated the distribution of Ki67 positive cells in actively inflamed and neoplastic regions of colons isolated from AOM/*Il10^−/−^* mice. Nuclear Ki67 staining was mostly restricted to the crypt bases in WT mice (Fig. 5A). In contrast, the colonic mucosa from inflamed *Il10^−/−^* mice showed areas of increased proliferation extending in some cases the full crypt length (Fig. 5A). In addition, colorectal adenomas harvested from *Il10^−/−^* mice showed consistently increased Ki67 staining compared to normal mucosa (Fig. 5B). This enhanced proliferation is consistent with increased accumulation of nuclear CTNNB1 in adenomatous tissues from *Il-10^−/−^* mice (Fig. 5C). Taken together, these data suggest that the heightened inflammatory and proliferative state observed in the context of the *Il10^−/−^* mouse colon coupled with AOM-induced activation of the WNT/CTNNB1 pathway results in an increased propensity for colorectal tumor formation and progression.

### Germ free *Il10^−/−^* mice mono-associated with *Bacteroides vulgatus* display reduced colitis and colon tumor development compared to *Il10^−/−^* SPF mice

**Figure 6:**
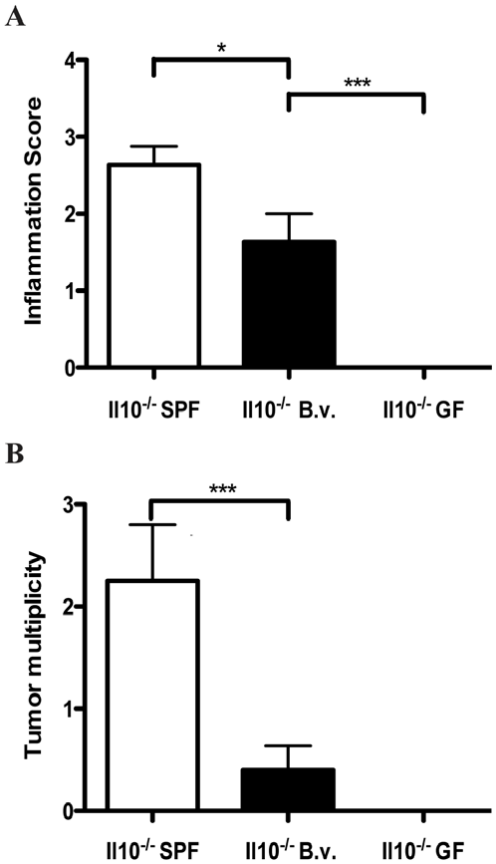
Modulation of microbiota-dependent colitis directly affects tumor development. A. Comparison of inflammation scores for *Il10^−/−^* mice under SPF, *B. vulgatus* mono-associated and germ free conditions. B. Tumor multiplicity in *Il10^−/−^* mice under SPF, *B. vulgatus* mono-associated and germ free conditions.

To further delineate the role of bacteria in promoting the development of CAC, we mono-associated germ-free WT and *Il10^−/−^* mice with *Bacteroides vulgatus* (*B. vulgatus*), an enteric bacterium, which has been reported previously to induce only mild colitis in the *Il10^−/−^* model of IBD [18]. Histological evaluation showed that *Il10^−/−^* mice colonized with *B. vulgatus* developed moderate colitis (average score = 1.6) compared to WT mice (average score = 0.36) (Fig. 6A). These values however were significantly less than inflammatory scores observed in conventionalized *Il10^−/−^* mice (average score = 2.6, p = 0.03) (Fig. 6A). Concordant with inflammation status, mono-associated *Il10^−/−^* mice displayed a significantly lower tumor multiplicity compared to their conventionalized counterparts (0.4 and 2.3 respectively, p = 0.002) (Figure 6B). Pearson linear regression analysis revealed a strong positive correlation between histological colitis score and colorectal tumor number among *B. vulgatus* mono-associated mice (p = 0.009). Moreover, AOM-treated germ free *Il10^−/−^* mice failed to develop colitis and consequently colorectal tumors as confirmed by histological evaluation (Fig. 6A & B). These findings indicate that the presence of colitogenic bacteria is essential for the development of CAC.

### MyD88 signaling is required for bacterial-induced CAC

**Figure 7:**
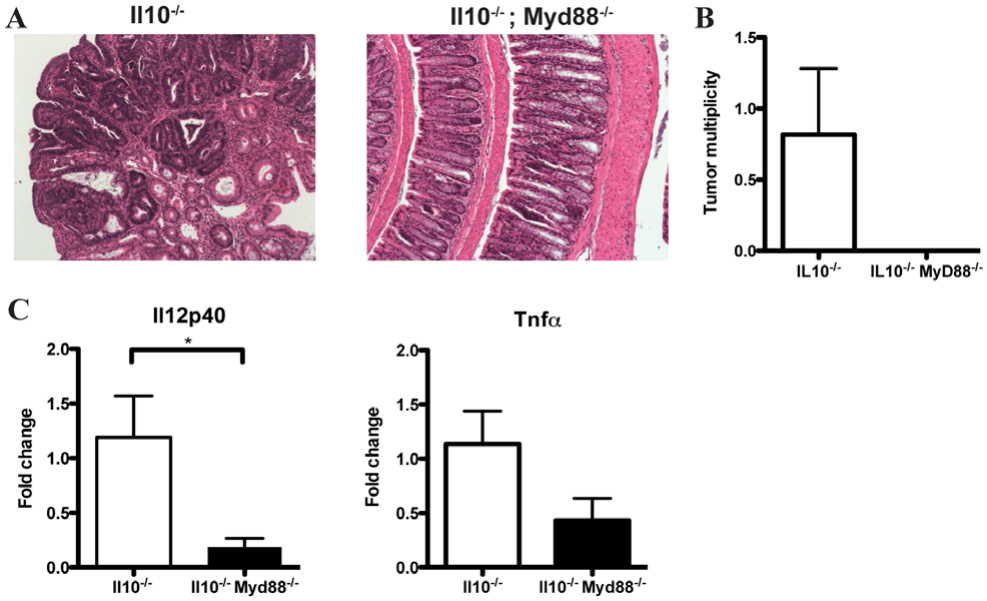
*Il10^−/−^*; *Myd88^−/−^* mice show decreased tumor multiplicity and expression of *Il12p40* and *Tnfα* mRNA. A. Representative histology observed in *Il10^−/−^* and *Il10^−/−^*; *Myd88^−/−^* mice treated with AOM. B. Tumor multiplicity in *Il10^−/−^* and *Il10^−/−^*; *Myd88^−/−^* mice treated with AOM. C. Relative expression of *Il12p40* and *Tnfα* mRNA in the distal colons of *Il10^−/−^* and *Il10^−/−^*; *Myd88^−/−^* mice.

To examine the role of TLR/MyD88 pathway signaling in CAC development, *Il10^−/−^*; *Myd88^−/−^* mice were administered AOM and colorectal tumor formation was evaluated. Colonoscopy showed the presence of neoplastic lesions in *Il10^−/−^* mice while *Il10^−/−^*; *Myd88^−/−^* mice showed no signs of disease (data not shown). Representative examples of colon tissue histology for *Il10^−/−^*and *Il10^−/−^*; *Myd88^−/−^* mice are shown in figure 7A. *Il10^−/−^* mice developed an average of 0.8 colorectal tumors/mouse while *Il10^−/−^*; *Myd88^−/−^* mice were devoid of neoplastic lesions (Figure 7B).

To explore the possibility that abrogated tumor formation in *Il10^−/−^*; *Myd88^−/−^* mice is the result of altered inflammatory-cytokine expression, we used semi-quantitative PCR to determine the relative mRNA expression levels of the pro-inflammatory cytokines *Tnf*α and *Il12p40* in the distal colon, the predominant site of tumor formation. We observed significantly lower *Il12p40* mRNA expression (p = 0.032) as well as a notable trend toward decreased *Tnfα* expression (p = 0.089) in *Il10^−/−^; Myd88^−/−^* compared to *Il10^−/−^* mice (Figure 7C). Altogether, these findings indicate that bacterial-induced inflammation promotes the development of colitis-associated colorectal cancer and is dependent on TLR/MyD88 pathway signaling.

## Discussion

Although UC represents the prototypical example of the link existing between chronic inflammation and risk of developing colorectal cancer, the mechanisms underlying this process remain to be elucidated. This is likely due to limitations posed by existing experimental models, which make it difficult to recapitulate the effects of chronic inflammation on colorectal tumorigenesis. In this study, we showed that colitogenic bacteria are necessary for the development of chronic intestinal inflammation leading to tumorigenesis. Using AOM as a tumor initiator and the microbiota to trigger chronic colitis in genetically susceptible *Il10^−/−^* mice, we show that inflammation directly correlates with colorectal tumor multiplicity and enhances tumor progression. These events were not observed in *Il10^−/−^* mice housed under germ-free conditions and were strongly attenuated in mice associated with *B. vulgatus*, a weak inducer of intestinal inflammation. Furthermore, disruption of MyD88 signaling, a key integrator of multiple TLRs prevented the development of colorectal tumors in *Il10^−/−^* mice. These findings strongly establish the microbiota as key in triggering intestinal inflammation and neoplastic changes in a susceptible host. The role of TLR signaling in the development of colorectal cancer has been the subject of recent intense investigation. Rakoff-Nahoum and co-workers showed that MyD88 signaling contributes to tumor progression in the *Apc^Min^* model of human familial adenomatous polyposis, suggesting a role for intestinal microorganisms in the process of tumorigensis [27]. However, because cytokines such as IL1 and IL18 also utilize MyD88 to activate downstream target genes [28] the involvement of the intestinal microbiota in this model is unclear. Moreover, the involvement of MyD88 signaling in the development of CAC was not addressed in this study. A better picture of the role of TLR signaling in CAC has begun to emerge from studies using the AOM/DSS model. For example, Fukata and coworkers demonstrated that colorectal tumorigenesis is strongly decreased in *Tlr4^−/−^* mice, suggesting that this innate receptor is important for the development of CAC [24]. However, this strong reduction in neoplasia was not accompanied by a concomitant reduction in inflammation. Consequently, there is an apparent disconnect between intestinal inflammation and tumorigenesis in the AOM/DSS model. This phenomenon is not specific to TLR signaling since a recent report showed that tumorigenesis is dramatically reduced in *Il6^−/−^* and *Stat3^−/−^* mice, despite significantly higher inflammatory scores in these mice [29]. In contrast, using the AOM/*Il10^−/−^* model, we observed a strong correlation between inflammation score and tumor multiplicity. This suggests that severe inflammation more aptly supports the survival of early transformation events. Additionally our data indicates that the presence of inflammation irrespective of severity influences tumor progression. It is possible that the tumor microenvironment present in the AOM/DSS model differs from that in the AOM/*Il10^−/−^* model, which could account for decreased tumorigenesis despite the high inflammatory state in the former model.

Recent reports have provided insight into the role of colitis as a promoter of colorectal tumorigenesis in the AOM/DSS model [24], [29]. However, one important question that remains unanswered is whether these tumor-promoting effects are elicited by the wound-healing response inherently caused by DSS treatment or by the inflammation that accompanies it. For example, Grivennikov and co-workers demonstrate that *Il6^−/−^* mice exhibit decreased tumor multiplicity and load compared to WT mice after AOM/DSS treatment yet these mice displayed more severe intestinal inflammation than WT mice. Consequently, reduced tumorigenesis in this model is the result of impaired IL6/STAT3 signaling in intestinal epithelial cells (IEC) and not necessarily of intestinal inflammation. We demonstrate here that the AOM/*Il10^−/−^* model of CAC can be used to investigate specifically, the impact of colitis on colorectal tumorigenesis. Whether IL6/STAT3 signaling is important in the development of CAC in AOM/*Il10^−/−^* mice remains to be investigated. Additionally, we show that CAC occurs in the context of activated NFκB pathway signaling as shown by the presence of NFκB positive immune cells suggesting that this inflammatory signaling pathway is important in the development and/or promotion of neoplasia. Additional studies will be required to more specifically define a role for NFκB-mediated inflammatory signaling in CAC.

In summary, we show that the gut microbiota is essential to the development of CAC and that chronic colitis promotes the oncogenic potential of colorectal tumors resulting in progression to advanced stages. These events are dependent on microbial recognition by the TLR/MyD88 system. Modulation of this important innate sensing system could represent a novel means by which to prevent/attenuate development of CAC.

## Materials and Methods

### Ethics statement

All animal protocols were approved by the Institutional Animal Care and Use Committee of the University of North Carolina at Chapel Hill.

### Mice and induction of CAC

WT and *Il10^−/−^* mice on the 129 SvEv background were bred and housed in the Gnotobiotic Animal Facility at the University of North Carolina at Chapel Hill. Ten to twelve week-old germ-free mice were transferred to specific pathogen free (SPF) conditions (conventionalized), and after 5 weeks were injected i.p. with 10 mg/kg body weight AOM (Sigma Aldrich) once a week for 6 weeks. *Il10^−/−^*; *Myd88^−/−^* mice and control *Il10^−/−^* mice (C57BL/6J/129) were raised under SPF conditions and at 12 weeks of age were injected with AOM as described above. All mice tested free of helicobacter hepaticus and helicobacter pylori, two known inducers of tumorigenesis in *Il10^−/−^* mice [30].

### Bacterial colonization

*Il10^−/−^*- and WT mice were mono-associated at 10–12 weeks of age by gavage feeding and rectal swabbing with viable bacteria cultured from guinea pig isolates of *Bacteroides vulgatus*. Mono-associated mice were maintained in the Gnotobiotic Animal Facility at the University of North Carolina at Chapel Hill. Bacterial association and absence of contamination by other bacterial species were confirmed by periodic aerobic culture of stool samples and gram staining.

### Necropsy and tumor histology

Upon sacrifice, colons were removed from the cecum to the rectum, flushed with PBS, splayed longitudinally and tumors counted. Distal colon tissue samples were collected and snap frozen. Colons were swiss-rolled from the distal to the proximal end, fixed overnight in 10% formalin and paraffin-embedded. Six µm sections were prepared and stained with hematoxylin and eosin for histologic analysis. Tumors were scored for inflammation and tumor grade.

Histological evaluation of mucosal inflammation was performed using a scoring system of 0 to 4 to classify the degree of lamina propria mononuclear cell infiltration, crypt hyperplasia, goblet cell depletion and architectural distortion, as previously described [18]. Tumors were classified as low-grade dysplasia when they displayed a disorganized epithelium lined by hyperchromatic cells with nuclear pseudostratification. Tumors were classified as high-grade when they contained back-to-back glands, high nuclear to cytoplasmic ratios, increased nuclear pleomorphism and loss of cellular polarity. Tumors were classified as invasive carcinomas when there was clear penetration of the dysplastic region through the muscularis mucosa resulting in desmoplastic response from the neighboring stroma. Inflammation and neoplastic lesions were scored by a trained pathologist.

### Immunohistochemistry

Tissue sections were deparaffinized in xylene and rehydrated through a graded series of alcohol washes. Immunohistochemistry for phosphorylated-Rel A (RelA 276) was performed with a rabbit polyclonal antibody diluted 1∶50 (Cell Signaling Technology) using the Vectastain Elite Rabbit IgG kit (Vector Laboratories). Ki67 immunohistochemisry was performed as follows: Endogenous peroxidase activity was blocked by incubating in 0.3% H_2_O_2_ for 30 minutes. Antigen retrieval was carried out by boiling sections for 7 minutes in 0.01 M citrate buffer pH 6.0 and cooled at room temperature for 30 minutes. Blocking was performed with 3% BSA in phosphate buffered saline (PBS) for 30 minutes at room temperature. Ki67 monoclonoal antibody (Dako) was diluted 1∶200. Biotinylated secondary antibody (Vector Laboratories) was diluted 1∶200 in 1% BSA/PBS. Subsequent steps were carried out using the Vectastain ABC kit according to manufacturer instructions. CTNNB1 immunohistohistochemistry was performed using a mouse anti-CTNNB1 antibody diluted 1∶100 (Transduction Laboratories) with the M.O.M. peroxidase kit (Vector Laboratories). Primary antibody incubation steps were carried out over-night at 4°C. Visualization was performed using 3,3′-diaminobenzidine (Dako).

### Real-time PCR

RNA was isolated from distal colon tissues using the TRIzol method (Invitrogen, Carlsbad, CA). cDNA was prepared by reverse transcribing 1 µg RNA using 126 units of M-MLV reverse transcriptase, 1 mM dNTPs and 875 pmol random primers (Invitrogen). Semi-quantitative real-time PCR was performed using an Eppendorf Realplex Master Cycler. PCR reaction: 150 nM final concentration of forward and reverse primers, 6 µl of QuantiTect SYBR Green PCR Master Mix (Qiagen, Valencia, CA) and 50 ng of cDNA template in a total of 12 µl. The following PCR conditions were used: 95°C, 15 minutes; (95°C, 15 seconds; 56°C, 30 seconds; 72°C, 30 seconds)×40 cycles. Specificity and linearity of amplification for each primer set was determined by melting curve analysis and calculation of the slope from serial diluted samples. Relative fold-changes were determined using the ΔΔCT calculation method. Values were normalized to the internal control β-actin. Primers: *Tnfα* (5′ ATGAGCACAGAAAGCATGATC 3′and 5′ TACAGGCTTGTCACTCGAATT 3′) and *Il12p40* (5′ CACGGCAGCAGAATAAATATG 3′ and 5′ TTGCATTGGACTTCGGTAGA 3′).

### Statistical analysis

Statistical analyses were performed using GraphPad Prism version 5.0a. Comparisons made between WT and *Il10^−/−^* or *Il10^−/−^* and *Il10^−/−^*; *Myd88^−/−^* mice were analyzed using a two-tailed unpaired t-test. Comparisons made between SPF, *B. vulgatus* mono-associated and germ-free mice were analyzed using a one-way analysis of variance (ANOVA). Individual comparisons were subsequently made using a two-tailed unpaired t-test. Pearson correlation analysis was performed to assess correlation between tumor number and inflammation severity in AOM-treated WT and *Il10^−/−^* mice (n = 22) using SAS (SAS Institute, Cary NC).

### Colonoscopy

Colonoscopy was performed 4, 8, 12, 16 and 18 weeks after transfer of mice from germ-free to SPF conditions (Fig. 1) to follow the progression of colitis and tumor formation. *In vivo* visualization of tumors was performed using a “Coloview System” (Karl Storz Veterinary Endoscopy). Mice were anesthetized using 1.5% to 2% isoflurane and ∼4 cm of the colon from the anal verge from the splenic flecture was visualized. The procedures were digitally recorded on an AIDA Compaq PC.
